# Blunted heart rate recovery is associated with coronary artery spasm in patients with suspected vasospastic angina

**DOI:** 10.1186/s40885-017-0080-2

**Published:** 2017-12-12

**Authors:** Hyunsu Kim, Sang-Hoon Cho, Kyoung-Im Cho, Bong-Joon Kim, Sung-Il Im, Jung-Ho Heo

**Affiliations:** 1 Division of Cardiology, Department of Internal Medicine, Kosin University College of Medicine, 34, Amnam-dong, Seo-gu, Busan, 602-702 Korea; 2 Department of Statistics and Actuarial Science, Soongsil University, Seoul, Korea

**Keywords:** Coronary artery spasm, Cardiac autonomic function, Heart rate recovery

## Abstract

**Background:**

Autonomic nervous system activity has been shown to be altered in patients with vasospastic angina (VA). Heart rate recovery (HRR) is a simple, non-invasive measurement of autonomic nervous system dysfunction. We aimed to investigate whether HRR is related to VA, as established by an ergonovine test.

**Methods:**

A total of 976 consecutive patients (47.5% male, mean age 55 years) without significant coronary artery disease who underwent both an ergonovine provocation test and a treadmill exercise test were enrolled. The relationship between VA and HRR was evaluated.

**Results:**

A total of 30.7% (300/976) of patients were diagnosed with VA, as documented by the ergonovine provocation test. HRR was significantly reduced in patients with VA compared to patients without VA (24.6 ± 18.0 vs. 30.5 ± 22.2, *p* < 0.001), and HRR was lowest in patients with multi-vessel spasm (21.9 ± 17.3). The proportion of blunted HRR, which was defined as HRR less than 12 beats, was significantly higher in patients with VA than in those without coronary artery spasm (26.6% vs. 39.3%, *p* < 0.001). In multivariable regression analyses, age (odds ratio (OR) = 1.03; 95% confidence interval (CI): 1.01–1.04; *p* = 0.001), blunted HRR (OR = 1.71; 95% CI: 1.26–2.31; *p* < 0.001), current smoking status (OR = 2.11; 95% CI: 1.50–2.98; *p* < 0.001), and male gender (OR = 2.00; 95% CI: 1.43–2.78; *p* < 0.001) were significant independent predictors of VA presence.

**Conclusion:**

Blunted HRR was an independent predictor of VA presence, which suggests a link between coronary artery spasm and autonomic dysregulation.

## Background

Coronary artery spasm, which is defined as transient occlusive or subocclusive vasoconstriction in large epicardial arteries, plays an important role in vasospastic angina (VA). Although the precise mechanism of VA is not well defined, one of the major causes of VA has been reported to be endothelial dysfunction of the coronary artery, which is characterized by impaired nitric oxide release [1]. Another important factor may be altered autonomic nervous activity, as changes in autonomic tone are likely to contribute to epicardial spasm [2]. However, because stimulation of both the sympathetic and parasympathetic nervous systems can provoke coronary artery spasms [2–4], the precise mechanisms by which spasms are triggered have not been elucidated.

Heart rate recovery (HRR) is a simple, non-invasive measurement of autonomic nervous system dysfunction, which is indicated by impaired parasympathetic reactivation [5–7]. The fast component seems to be mainly dictated by cardiac parasympathetic reactivation, while the slow phase of HRR may be determined by cardiac sympathetic withdrawal [8]. HRR after exercise is emerging as a new and important prognostic index [9, 10], and an earlier study showed that a blunted HRR, which is defined as a decrease in heart rate (HR) of less than 12 beats/min from peak exercise to 1 min into recovery, is a powerful predictor of overall mortality [5, 11]. Although autonomic nervous system activity is thought to play an important role in the initiation of coronary spasm, it is unclear to what extent the spasm is associated with abnormal autonomic nervous activity. Moreover, the association between coronary artery spasm and autonomic function assessed by HRR has not been well studied. Therefore, the purpose of this study was to investigate whether HRR is related to VA, as established by an ergonovine test.

## Methods

This cross-sectional, observational, single-center, cohort study included 1536 consecutive patients with stable angina, exclusively or predominantly induced by effort who underwent coronary angiogram with an ergonovine provocation test (EPT) and an exercise treadmill test from January 2007 to August 2015. The standard medications such as beta blockers, nitrate and calcium channer blockers were withheld during the exercise treadmill test and EPT. Patients were excluded from the study if any of the following criteria were met: any systemic disease, such as significant liver disease, neurologic disorders, or malignant disease, secondary hypertension, valvular heart disease, significant coronary stenosis (≥50%) on coronary angiogram, atrial fibrillation, history of heart failure, history of acute coronary syndrome, previous myocardial infarction, or previous revascularization procedure. Demographic characteristics recorded at the first visit included age, sex, height, weight, current medications, smoking history and alcohol consumption. Blood was drawn to measure total serum cholesterol, triglycerides, high-density lipoprotein (HDL) and low-density lipoprotein (LDL) cholesterol, blood glucose, creatinine, uric acid, and high sensitivity C-reactive protein (hs-CRP). Body mass index (BMI) was calculated as the ratio of dry weight in kilograms to the square of height in meters.

### Exercise treadmill testing

On the same day as the echocardiographic examination, patients underwent symptom-limited exercise stress testing (GE CASE T2100; GE Medical Systems, Milwaukee, WI, USA) according to the protocol by Bruce et al. [12] Blood pressure (BP) was measured with an automated BP monitor (Suntech Tango; Suntech Medical, Morrisville, NC, USA) throughout the treadmill test using the same arm on which resting BP was measured. A 12-lead electrocardiograph was monitored continuously and was printed at a paper speed of 25 mm/s. HR and BP measurements were recorded at the end of each three-minute stage, at peak exercise, and at one-minute intervals throughout recovery. The participants continued to exercise until becoming fatigued or if HR exceeded 85% of the estimated maximum HR (220 bpm - age). Total exercise time was also recorded. Functional capacity was estimated in metabolic equivalents (METs) on the basis of treadmill speed and grade [13]. During the recovery phase, the subjects continued to walk for 60 s at a speed of 1.5 mph, and then sat down for 3 min with continued monitoring of BP, HR, and heart rhythm. The HRR value was defined as the decrease in HR from peak exercise to 1 min after the cessation of exercise. An abnormal HRR value was defined as a decrease of less than 12 beats/min, in accordance with a previous study [11].

### Ergonovine test for provocation of coronary spasm

Patients with normal angiography or minimal luminal narrowing (<50% stenosis) underwent an ergonovine provocation test. The ergonovine provocation test was performed with intravenous injection of methylergometrine maleate. After performing a diagnostic coronary angiography, incremental doses of ergonovine were injected intravenously (50, 100, 200 μg) over 10 s [14]. Two minutes after each injection, coronary angiogram, electrocardiogram, blood pressure, and patient symptoms were assessed. A positive ergonovine test was defined as near total occlusion or localized spasm (≥90% diameter) of the epicardial coronary artery with signs of chest pain or ischemic ST changes according to the Guidelines for Diagnosis and Treatment of Patients with Vasospastic Angina [15, 16]. An intracoronary injection of isosorbide dinitrate was administered upon completion of the ergonovine test, regardless of whether a coronary spasm was confirmed. We continuously assessed the coronary angiogram and electrocardiogram for changes in vessel diameter and electrophysiology. No patients had histories of myocardial infarction, congestive heart failure, previous coronary stenting, or other serious diseases. We divided the study patients into a VA group, which included patients who showed definite coronary spasm on the ergonovine test, and a non-VA group, which included those who did not show typical vasoconstriction. We defined multi-vessel coronary artery spasm as spasm in two or three of the three major coronary arteries (right, left anterior descending, or left circumflex coronary artery).

### Statistical analyses

Any significant differences between the with- and without-VA groups were tested using t-statistics for continuous data or chi-square statistics for categorical data. The association between VA and clinical and laboratory variables, including HRR, was investigated using logistic regression models. To prevent over-fitting and multicollinearity, variable selection was performed in a stepwise fashion. The calibration and discriminability of logistic regression models were validated by the Hosmer-Lemeshow test and the C-index, respectively. Also, the goodness-of-fit and discriminability of four different logistic regression models were compared using likelihood ratio tests and Delong’s tests. Model 1 includes conventional cardiovascular risk factors such as age, gender, and smoking status; Model 2 extends Model 1 to include maximum heart rate; Model 3 extends Model 2 to include blunted heart rate recovery. For all tests, the significance level was set to *p* ≤ 0.05. All statistical analyses were performed using R (http://www.r-project.org).

## Results

### Clinical characteristics of the study population (Table 1)

A total of 30.7% (300/976) of patients were diagnosed with VA by the ergonovine provocation test. Baseline clinical and laboratory characteristics according to VA presence are shown in Table 1. Compared to patients without VA (*n* = 676), those with VA (*n* = 300) were more likely to be male (41.1% vs. 62%, *p* < 0.001), current smokers (23.5% vs. 45%, *p* < 0.001), and alcohol drinkers (32% vs. 41.3%, *p* = 0.006). Furthermore, the VA group had a higher incidence of dyslipidemia (42.5% vs. 52%, *p* = 0.007) and trend of diabetes mellitus (11.6% vs. 16.3%, *p* = 0.052). Age, BMI, BP, HR, and the number of patients taking calcium antagonists were similar between the groups.

**Table 1:** Baseline clinical and laboratory characteristics according to the presence of vasospastic angina (VA)

	Patients without VA (*n* = 676)	Patients with VA (*n* = 300)	*p*-value
Age, years	53.2 ± 10.9	55.7 ± 9.8	<0.001
Male gender, n (%)	278 (41.1%)	186 (62%)	<0.001
Body mass index, kg/m^2^	24.6 ± 3.3	24.7 ± 3.2	0.681
systolic BP, mmHg	147.3 ± 24.1	148.2 ± 24.1	0.568
diastolic BP, mmHg	74.1 ± 13.2	74.3 ± 12.4	0.784
Current smoking, n (%)	159 (23.5%)	135 (45%)	<0.001
Alcohol drinker, n (%)	216 (32%)	124 (41.3%)	0.006
Diabetes mellitus, n (%)	78 (11.6%)	49 (16.3%)	0.052
Dyslipidemia, n (%)	287 (42.5%)	156 (52%)	0.007
Hypertension, n (%)	200 (29.6%)	90 (30%)	0.956
Hyperthyroidism, n (%)	28 (7.5%)	8 (5.3%)	0.466
Previous medication			
Aspirin	106 (15.8%)	44 (14.7%)	0.752
RAS blocker	89 (13.2%)	52 (17.3%)	0.109
Beta blocker	67 (9.9%)	36 (12%)	0.390
Calcium channel blocker	91 (13.5%)	45 (15%)	0.601
Diuretics	19 (2.8%)	9 (3%)	1.000
Nitrates	65 (9.7%)	44 (14.7%)	0.029
Statin	83 (12.4%)	57 (19%)	0.009
Uric acid, mg/L	5.4 ± 1.5	5.5 ± 1.5	0.492
Creatinine, mg/dL	0.8 ± 0.2	0.8 ± 0.2	0.973
Fasting glucose, mg/dL	101.9 ± 24.9	98.6 ± 19.5	0.054
Total cholesterol, mg/dL	176.5 ± 37.1	174.2 ± 35.3	0.375
LDLcholesterol, mg/dL	105.2 ± 36.0	103.1 ± 31.2	0.391
HDL cholesterol, mg/dL	47.7 ± 14.5	47.3 ± 13.0	0.647
Triglycerides, mg/dL	124.7 ± 120.1	131.6 ± 103.4	0.386
hs-CRP, mg/dL	0.3 ± 1.0	0.3 ± 1.3	0.973

All values are means ± SDs for continuous variables or frequencies (percentages) for categorical variables

*BP* blood pressure, *RAS* renin angiotensin system, *LDL* low density lipoprotein, *HDL* high density lipoprotein, *hs-CRP* high sensitivity C-reactive protein

### Comparisons of exercise stress testing according to presence of coronary spasm

Table 2 shows the comparison of symptom-limited exercise stress testing according to VA presence. There were no significant differences in left ventricular ejection fraction, exercise time, or metabolic equivalents between the groups. However, HRR was significantly reduced in patients with VA compared to those without VA (24.6 ± 18.0 vs. 30.5 ± 22.2, *p* < 0.001). The proportion of blunted HRR was significantly higher in patients with VA than in those without VA (39.3% vs. 26.6%, *p* < 0.001). When we analyzed HRR according to the number of vessel with spasm regarding multiple spasm (> = 2 vessels), HRR was significantly reduced in multiple spasm compared to the single spasm (21.9 ± 17.3 vs. 27.6 ± 18.1, *p* = 0.02). When we analyzed HRR according to the lesion site, HRR showed no significant difference between diffuse spasm and focal spasm (28.6 ± 21.5 vs. 27.9 ± 18.6, *p* = 0.61).

**Table 2:** Comparison of symptom-limited exercise stress testing according to the presence of vasospastic angina (VA)

	Patients without VA (*n* = 676)	Patients with VA (*n* = 300)	*p*-value
Left ventricular ejection fraction, %	68.1 ± 7.8	68.4 ± 6.6	0.556
Exercise time, min	8.8 ± 2.2	8.7 ± 2.3	0.358
Metabolic equivalents	9.8 ± 4.3	9.4 ± 2.3	0.082
Rest heart rate, bpm	70.5 ± 12.7	67.8 ± 11.6	0.002
Max heart rate, bpm	152.1 ± 22.4	145.5 ± 21.2	<0.001
HRR, bpm	30.5 ± 22.2	24.6 ± 18.0	<0.001
Blunted HRR	180 (26.6%)	118 (39.3%)	<0.001
Rest systolic BP, mmHg	125.9 ± 13.2	126.9 ± 12.2	0.243
Rest diastolic BP, mmHg	78.0 ± 8.9	78.6 ± 9.1	0.394
Max systolic BP, mmHg	170.0 ± 17.5	169.1 ± 17.3	0.474
Max diastolic BP, mmHg	81.8 ± 10.3	80.8 ± 10.3	0.168

All values are means ± SDs for continuous variables or frequencies (percentages) for categorical variables

*HRR* heart rate recovery, *BP* blood pressure

### Prognostic value of blunted HRR and max HR

According to simple logistic regression analyses, marginally significant variables were age, gender, resting heart rate, smoking status, alcohol status, diabetes mellitus, dyslipidemia, nitrates, statin, max HR, blunted HRR, and HRR (Table 3), which were included as covariates into a multivariable logistic regression model.). In multivariable regression analyses, age (odds ratio (OR) = 1.03; 95% confidence interval (CI): 1.01–1.04; *p* = 0.001), blunted HRR (OR = 1.71; 95% CI: 1.26–2.31; *p* < 0.001), current smoking status (OR = 2.11; 95% CI: 1.50–2.98; *p* < 0.001), and male gender (OR = 2.00; 95% CI: 1.43–2.78; *p* < 0.001) were significant independent predictors of VA presence (Table 3).

**Table 3:** Risk factors significantly associated with vasospastic angina (VA) according to logistic regression models

Risk factors	Simple				Multivariable			
	Odds ratio	95% CI		p-value	Odds ratio	95% CI		p-value
		lower	upper			lower	upper	
Age	1.02	1.04	1.04	0.001	1.03	1.01	1.04	0.001
Male gender	2.34	1.77	3.09	<0.001	2.00	1.43	2.78	<0.001
Rest heart rate	0.98	0.99	0.99	0.002				
Current smoking	2.66	3.55	3.55	<0.001	2.11	1.50	2.98	<0.001
Alcohol drinker	1.50	1.99	1.99	0.005				
Diabetes mellitus	1.49	1.13	2.19	0.042				
Dyslipidemia	1.46	1.01	1.93	0.006				
Nitrates	1.61	1.06	2.41	0.023				
Statin	1.66	1.15	2.4	0.007				
Max heart rate	0.99	0.98	0.99	<0.001	0.99	0.98	1.00	0.005
Blunted HRR	1.79	1.34	2.38	<0.001	1.71	1.26	2.31	<0.001
HRR	0.99	0.98	0.99	<0.001				

*CI* confidence interval, *HRR* heart rate recovery

According to stepwise model selection, both max HR (adjusted OR = 0.99; 95% CI: 0.98–1.00; *p* = 0.005) and blunted HRR (adjusted OR = 1.71; 95% CI: 1.26–2.31; *p* = 0.001) were independent covariates significantly associated with VA after adjusting for conventional cardiovascular risk factors, including age, gender, and smoking status (Table 4).

**Table 4:** Incremental values of blunted heart rate recovery and max heart rate in terms of the goodness-of-fit and discriminability compared with conventional clinical prognostic factors

	Likelihood ratio test			Discriminability		
	LogLik	Diff.(%)	*p*-value^*^	C-index (%)	Diff.(%)	*p*-value^+^
Model 1	−562.08			66.70		
Model 2	−557.39			68.32		
Model 3	−551.50			69.34		
Model 1 vs. Model 2		4.69	0.002		1.63	0.015
Model 1 vs. Model 3		10.58	<0.001		2.64	0.007

Model 1: age + gender + current smoking; Model 2: Model 1 + max heart rate; Model 3: Model 2 + blunted heart rate recovery; *LogLik* loglikelihood, *Diff* difference, *c-index* Harrell’s concordance index; *p*-value^*^ is based on the loglikelihood ratio test; *p*-value^+^ is based on the Delong’s test

### Incremental effects of blunted HRR and max HR

**Fig. 1:**
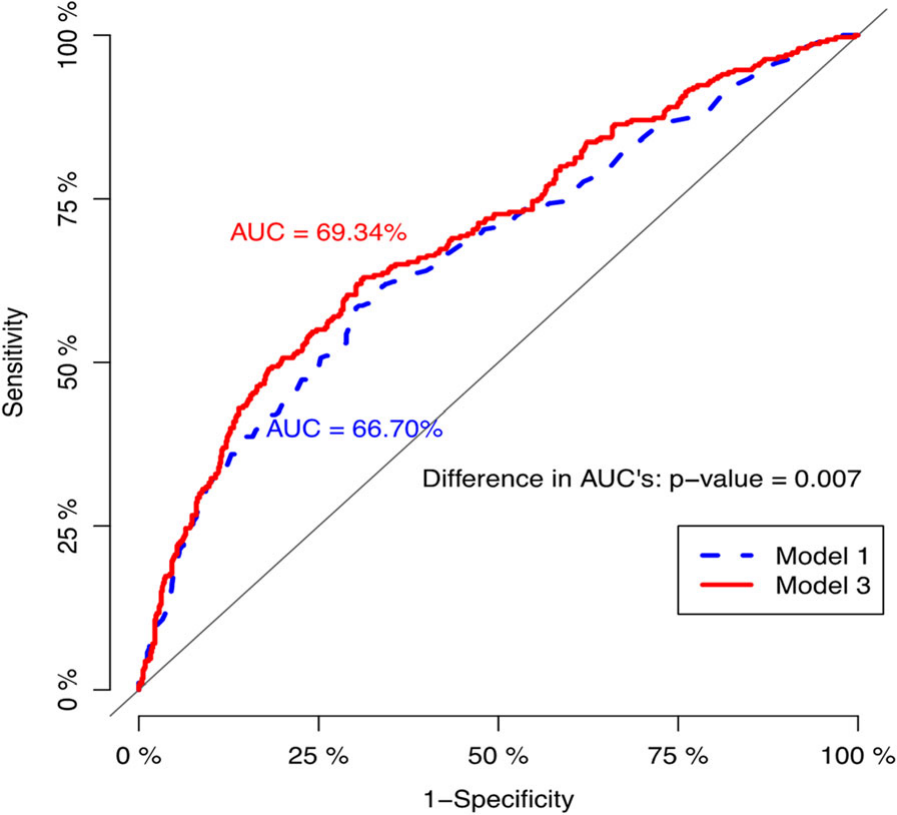
Comparison of areas under the curve (AUCs) of logistic regression models. On the receiving operator characteristic analysis, blunted HRR together with max HR significantly improved the goodness-of-fit and discriminability of Model 1 (log-likelihood difference = 10.58; *p* < 0.001; C-index difference = 2.64%; *p* = 0.007). Model 1: age + gender + current smoking; Model 3: Model 1 + max heart rate + blunted heart rate recovery

We evaluated the incremental effects of max HR and blunted HRR on VA by comparing three different logistic regression models (Table 4). According to comparisons among these models, blunted HRR together with max HR significantly improved the goodness-of-fit and discriminability of Model 1 (log-likelihood difference = 10.58; *p* < 0.001; C-index difference = 2.64%; *p* = 0.007; Fig. 1).

## Discussion

To the best of our knowledge, this is the first study to investigate the association between coronary artery spasm and HRR using symptom-limited exercise testing in VA patients. Most importantly, we found that: 1) HRR was lower in VA patients than non-VA patients; 2) there was a significant association between extent of coronary spasm and HRR; and 3) blunted HRR was an independent predictor of VA presence. Our findings suggest a possible link between coronary spasm and autonomic dysregulation in VA patients.

VA is an important cause of critical heart conditions such as acute myocardial infarction, fatal arrhythmia, and sudden cardiac death [17, 18]. Therefore, it is important to predict the risk of coronary artery spasm in the general population to reduce the incidence of associated complications. Modulation of the autonomic nervous system is thought to be a mechanism of VA. A previous study showed that a reduction in sympathetic activity followed by enhanced vagal activity may play a key role in triggering spontaneous coronary spasm, and that the secondary activation of sympathetic activity may worsen coronary spasms, resulting in an attack [3]. In addition, because coronary spasm can be induced by intracoronary injection of acetylcholine, the neurotransmitter of the parasympathetic nervous system, changes in the activity of the autonomic nervous system may be involved in the circadian variation of coronary spasm. Coronary spasm can also be induced by stimulation of alpha-adrenergic receptors. In this study, we showed that HRR was associated with coronary spasm, as established by the ergonovine test and HRR was significantly reduced in multiple coronary vessel spasm compared to the single coronary vessel spasm. In previous study, impaired circardian rhythm of autonomic nervous activity might be responsible for coronary spasm, especially multivessel spasm [4]. Our finding suggests that autonomic dysregulation assessed by HRR also have a proportional relationship with coronary artery spasm. The presence of blunted HRR was significantly associated with increased coronary vasospasm in VA patients who had not experienced an anginal attack. Generally, the increase in HR during exercise occurs as a result of combined sympathetic activation and parasympathetic withdrawal. In contrast, parasympathetic reactivation is the principal determinant of decreased HR during early recovery. This mechanism is independent of age and intensity of exercise [19]. Given the prognostic significance of diminished parasympathetic tone at rest, post-exercise HRR is a noninvasive method that can be used to assess parasympathetic activation [19, 20]. Thus, it can be inferred that parasympathetic “insufficiency” is implicated in the increased mortality risk for patients with abnormal HRR [9, 10, 19, 21]. Because HRR is simple to calculate from data obtained from standard exercise tests and because it does not require 24-h Holter monitoring or baro-reflex sensitivity testing, HRR may be valuable for assessing risk in routine clinical practice.

Considering that vagal withdrawal may contribute to the mechanisms of spontaneous coronary vasospasm [2], and that decreased parasympathetic activity with enhanced sympathetic activity at night was found to be involved in multi-vessel coronary spasm [4], we propose a correlation between blunted HRR and severity of coronary spasm. Our results showed that HRR was lowest in patients with multi-vessel coronary spasms, and there was a significant correlation between HRR and spasm extent. These findings suggest that blunted parasympathetic tone might be the dominant feature of coronary spam. Moreover, blunted HRR was an independent predictor of a positive ergonovine test, which implicates a link between VA and autonomic dysregulation.

This study has several limitations. First, because we defined a positive ergonovine test as near total spasm or localized spasm that reproduces the patient’s typical symptoms or is associated with ST segment shifts, our results are not applicable to clinically suspected VA patients with mild generalized vasoconstriction (<50% narrowing) of apparently normal coronary arteries. Although we tried to regard definite VA according to the guideline as VA and others as without-VA, there may be an error in the group with approximately 90% stenosis. So, dichotomous analysis rather than quantitative analysis may be a limitation of this study. Second, the cross-sectional study design eliminated our ability to determine causal relationships. Third, because this study was performed at a single tertiary care center, there were potentially biases with respect to patient referral and population sampling. Fourth, our patients were treated by various medications that may have had some effect on endothelial function. Fifth, a weak association between blunted HRR and coronary vasospasm indicates a need for a large prospective study. In addition, we induced the coronary vasospasm with ergonovin because acetylcholine is not available to use in Korea. Finally, because diabetic mellitus is a well-known disease combined with autonomic disturbance, and including them may be problematic. When we performed sensitivity analysis, the result was not changed. Despite these limitations, our study results indicate that HRR could be a valuable measure for evaluating VA.

## Conclusion

In conclusion, we demonstrated that blunted HRR is significantly associated with increased coronary vasospasm. These results support the need to re-evaluate the impact of autonomic dysregulation of endothelial activity of VA patients. However, longitudinal studies with a large sample population will be needed to determine the pathophysiologic and prognostic implications of coronary vasospasm.
